# Enhancing insulin secretion by pancreatic β-cell redifferentiation: a study of the anti-diabetic effects of berberine *in vitro*

**DOI:** 10.3389/fendo.2025.1658671

**Published:** 2025-12-15

**Authors:** Jing-Jing Xing, Wei Li, Chen Chen

**Affiliations:** 1 School of Biomedical Sciences, University of Queensland, Brisbane, QLD, Australia; 2 School of Chinese Medicine, Jilin Agricultural University, Changchun, Jilin, China

**Keywords:** insulin, berberine, beta cell (β cell), differentiation, secretion, glucose stimulated insulin response

## Abstract

The β-cell dedifferentiation and impaired glucose-stimulated insulin secretion (GSIS) contribute to the pathogenesis of diabetes. Berberine (BBR), a natural compound with known anti-diabetic therapeutic properties, has been investigated for its potential role in preserving β-cell function. This study aims to elucidate the molecular mechanism by which BBR prevents β-cell dedifferentiation and restores deteriorated GSIS. In this *in vitro* study, the MIN-6 and INS-1 pancreatic β-cell lines were exposed to a FoxO1 inhibitor, excessive levels of H_2_O_2_ or free fatty acids (FFAs) to induce dedifferentiation and impaired GSIS. BBR treatment was administered to assess its impact on β-cell fate and function. Molecular analyses, including gene expression profiling and pathway analysis, were conducted to unravel the underlying molecular mechanisms. BBR treatment significantly reduced β-cell dedifferentiation induced by FoxO1 inhibitor, H_2_O_2_ and FFAs. BBR treatment effectively reversed the poor GSIS observed under the FoxO1 inhibition and high concentrations of H_2_O_2_ and FFAs, restored the ability of β cells to respond normally to glucose by secreting insulin. The protective effect was associated with the modulation of nuclear factor kappa B (NF-κB) signaling pathway, while BBR reduced the overproduction of NF-κB, which has been proved by applying NF-κB angoist in INS-1 cell line. This study explored the protective potential of BBR in preventing β-cell dedifferentiation and restoring impaired GSIS, under the conditions of FoxO1 inhibition, excessive levels of H_2_O_2_ and FFAs. BBR may therefore be a promising candidate for diabetes treatment. *In-vivo* experiments are warranted to evaluate dedifferentiation levels in rodent diabetic models and the efficacy of BBR on the pancreatic β-cell redifferentiation in diabetes.

## 1 Introduction

Diabetes mellitus (DM) is commonly caused by insufficient insulin secretion and β-cell mass (1, 2). However, long-term exposure to glucose, a phenomenon known as glucotoxicity, leads to the loss of β-cell mass and insulin content via increased β-cell dedifferentiation and apoptosis (3). Loss of terminal cell identity has been increasingly reported across various tissues in type 2 diabetes (T2D) (4). While β-cell mass was once thought to be regulated primarily by the balance between β-cell replication and apoptosis (5). It is now evident that β-cell dedifferentiation plays a major role in β-cell failure (6). Dedifferentiated β-cells exhibit a loss of key factors essential for mature adult β-cells, gain endocrine progenitor markers, and frequently express other islet cell hormones (3, 7, 8). Similar differentiation impairments have been identified in other tissues affected by T2D (9, 10). Markers such as Nanog and L-myc have also been identified in β-cells undergoing dedifferentiation (8). Glucose-stimulated insulin secretion (GSIS) from human islets may decline even without a measurable loss of β-cell number (17), suggesting that β-cell dedifferentiation may contribute to functional impairment in T2DM (8). It was reported that dedifferentiated cells accounted for 31.9% of β-cells in persons with type 2 diabetes vs an 8.7% in controls and affected all endocrine cells for 16.8% in T2DM vs 6.5% in controls (11). The number of ALDH1A3-positive (a biomarker of β-cell dedifferentiation), insulin-negative cells was threefold higher in individuals with diabetes.

Defective mitochondrial quality, including genome stability, dynamics, and turnover has been proposed to impair oxidative phosphorylation, activate the mitochondrial integrated stress response, induce chromatin remodelling, and promote cellular immaturity rather than apoptosis, ultimately contributing to metabolic dysfunction (12).

Elevated levels of FFAs, particularly saturated fatty acids, are known to induce lipotoxicity in β cells. This may lead to metabolic dysfunction, apoptosis, and dedifferentiation (13, 14). In addition to the lipotoxicity in β cells, H_2_O_2_, a reactive oxygen species (ROS), may induce oxidative stress and cellular damage. Chronic exposure to H_2_O_2_ caused apoptosis, while acute exposure alters signalling pathways to cause dedifferentiation (15). Therefore, developing preventive strategies to restore impaired β-cell function is urgently needed.

Berberine (BBR), a plant-derived isoquinoline alkaloid, has been clinically shown to improve glycaemic control and lipid profiles in patients with T2D and dyslipidaemia (16) (17). While several studies have demonstrated BBR’s potential benefits, such as anti-inflammatory, antioxidant, and metabolic regulatory effects, its precise mechanisms, especially in the context of β-cell dedifferentiation, remain insufficiently explored (18). Notably, most prior studies have focused on systemic effects or glucose-lowering mechanisms via hepatic or intestinal pathways. In contrast, the current study aims to dissect the direct cellular actions of BBR on pancreatic β-cells under metabolic stress. This study investigated the effects of FFAs and H_2_O_2_ on MIN-6 and INS-1cells with or without BBR to elucidate the specific pathways underlying BBR’s protective effects on β-cells. The primary functions of BBR were confirmed as anti-inflammation, antioxidant, through modulation of FoxO1 signal. In addition, BBR prevented the dedifferentiation caused by FFAs and H_2_O_2_, suggesting a novel therapeutic strategy for preserving β-cell differentiation and function in diabetes.

## 2 Materials and methods

### 2.1 Chemicals

BBR (purity ≥ 99%), determined by high-performance liquid chromatography (HPLC), was obtained from Sigma-Aldrich (St. Louis, MO, USA). Palmitate was obtained from Sigma (Sigma, St. Louis, MO). DMEM and other culture reagents were obtained from Sigma (St. Louis, MO). The cell culture plates were purchased from Nalge Nunc International (Roskilde, Denmark). MTT (3-(4,5-Dimethylthiazol-2-yl)-2,5-Diphenyltetrazolium Bromide) was obtained from ThermoFisher. VF647A-Annexin V/PI Apoptosis Detection Kit and FoxO-1 inhibitor (AS1842856) were purchased from MCE (MedChemExpress). The anti-Insulin and proinsulin antibody-D6C4 was obtained from Abcam (Abcam, ab8303). The mouse or rabbit polyclonal antibodies anti-ALDH1A3, anti-p-IKKα, IKKα, p-p65, p65; p-IkB, IkB, β-actin and GAPDH were obtained from Cell Signaling Technology (Beverly, MA). Secondary antibodies were purchased from Proteintech (USA). Materials for rat insulin RIA were obtained from Linco Research (St. Charles, MO).

### 2.2 Cell culture and treatment protocols

Islet β-cells INS-1 and MIN-6 cells were maintained in DMEM supplemented with 10% FBS, 1% penicillin-streptomycin (PS) and 1% β-mercaptoethanol in an atmosphere of 5% CO_2_ at 37 °C. Cells were routinely passaged and were resuspended by Trypsin-EDTA. MIN-6 cells were seeded in 96 well plates at a density of 1 × 10^6^ cells/well or in six well plates at a density of 10^5^ cells/well. DMEM (containing 10% FBS and 1% β-mercaptoethanol) without 1% penicillin-streptomycin was used to culture the cells. All experiments with MIN6 cells were performed between passages 21 and 28.

MIN-6 or INS-1 cells were treated with FoxO1 inhibitor for 24-hour or 48-hour to induce the dedifferentiation, and BBR was treated for another 24-hour or 48-hour. After treatment, cells were washed by PBS or glucose-free modified Krebs-Ringer bicarbonate (KRB) buffer containing 5 mmol/l NaHCO_3_, 1 mmol/l CaCl_2_, 0.5% (wt/vol) BSA, and 10 mmol/l HEPES (pH 7.4) for GSIS testing. After testing, cells were collected for western blot or RT-PCR. For the oxidative stress induced MIN-6 dedifferentiation, BBR was pretreated for 24 hours. Hydrogen peroxide pretreatment was done by incubating MIN-6 cells with 2.5 μM H_2_O_2_ for 30 min, followed by washing twice with PBS or KRB buffer. Free fatty acid mixture (palmitate) was prepared as previously described (19, 20). The ratio between BSA and FA is 3:1 (molar ratio). Firstly, palmitate was dissolved in 50% ethanol to a concentration of 100 mM, which was then diluted in DMEM to a various concentrations and then mixed with 0.5% fatty acid free BSA (Sigma) for 30 min at 37°C. Cells were exposed to palmitate for 24 hours in the presence of different concentrations of BBR for 24 hours.

### 2.3 MTT assays and treatment of BBR

MIN-6 cells were seeded into 96 well plates. When the cell confluency or cell density reached approximately 80%, the control group, FFAs (0.04 and 0.08 μM) treatment, H_2_O_2_ (2.5 µM) or FoxO1 inhibitor (5 µM) groups, combined with different concentrations of BBR (2, and 4 μM) 24-hour treatment groups were established accordingly. The cell viability was measured by the MTT method after 24-hour of cell culture, and the optimal BBR concentration was selected for the three models. The criteria for the selected concentrations were: 1. not affecting cell proliferation or apoptosis; 2, the lowest concentration with an optimized effectiveness. When cells were ready for analysis, MTT reagent (5 mg/ml MTT in PBS) at 37°C was added into culture plate for 3 hours. After incubation, cells were treated with 150 μl dimethyl sulfoxide (DMSO), then the culture plate was placed on a shaker and shaken at low speed for 5 mins to fully dissolve the crystals. The absorbance of each well at 570 nm wavelength (Tecan) was measured as MTT assay result.

### 2.4 Cell apoptosis assay

Cell apoptosis was measured by flow cytometry analysis. MIN-6 cells were cultured in DMEM with 10% FBS alone or with additional BBR at various concentrations for 24 hours or 48 hours. Cells were then harvested at a density of 3 × 10^6^ cells/mL in the binding buffer and stained with the Annexin V/propidium iodide (PI) staining assay (BD Biosciences, San Jose, CA, USA) for 5 min at room temperature and were analysed using a FACS Calibur flow cytometer and Cell Quest software (BD Biosciences). Apoptotic cells were identified by Annexin V+ staining and subdivided into early apoptotic cells (PI−/Annexin V+) or late apoptotic cells (PI+/Annexin V+).

### 2.5 Assessment of cell proliferation using CM20 assay

Cell proliferation was assessed using the CM20 automated cell monitoring system (Nikon, Japan), which allows real-time, non-invasive tracking of cell growth in culture. MIN-6 cells were seeded into 96-well plates and cultured until they reached approximately 60–70% confluence. Cells were then treated with various concentrations of BBR and monitored over a 24- to 48-hour period. The CM20 system automatically scanned the wells at regular intervals, capturing high-resolution images to quantify both cell number and confluency. These parameters were analyzed to evaluate the effect of BBR on cell proliferation. At the end of the monitoring period, all data were transferred to a computer and analyzed using GraphPad Prism 10 software.

### 2.6 Quantitative real time PCR

Total RNA was extracted from treated cell samples using the PureLink™ RNA Mini Kit (Thermo Fisher Scientific) or TRIzol™ Reagent (Invitrogen), following the manufacturers’ protocols. RNA purity and concentration were assessed using a NanoDrop™ spectrophotometer (Thermo Fisher Scientific). First-strand complementary DNA (cDNA) was synthesized from 1 µg of total RNA using the iScript™ Reverse Transcription Supermix (Bio-Rad) according to the manufacturer’s instructions. Quantitative real-time PCR (qPCR) was performed using iTaq™ Universal SYBR^®^ Green Supermix (Bio-Rad) on a CFX96™ Real-Time PCR Detection System (Bio-Rad). Each 20 µL reaction contained 10 µL SYBR Green Supermix, 1 µL of cDNA template, 0.5 µL of each forward and reverse primer (10 µM), and nuclease-free water. The amplification protocol consisted of an initial denaturation at 95°C for 2 minutes, followed by 40 cycles of 95 °C for 5 seconds and 60°C for 10 seconds. Each sample was run in technical triplicate. β-actin (Actb) was used as the internal control for normalization. Non-reverse transcriptase (No-RT) and no-template control (NTC) reactions were included on each plate to rule out genomic DNA contamination and non-specific amplification. Relative gene expression was calculated using the 2^−ΔΔCt method. Data were analyzed using GraphPad Prism 10 (GraphPad Software). The primers used were listed as following: INS-2, FWD: CGTGGCTTCTTCTACACACCCA, RVS: TCCAGTGCCAAGGTCTGAAGGT; Nkx2.1, FWD: CAGGACACCATGCGGAACAGC; RVS: GCCATGTTCTTGCTCACGTCCC; MafA, FWD: GCTTCAGCAAGGAGGAGGTCAT; RVS: TCTCGCTCTCCAGAATGTGCCG; FoxO1, FWD: CTACGAGTGGATGGTGAAGAGC; RVS: CCAGTTCCTTCATTCTGCACTCG.

### 2.7 Western blot

Cells treated in 6 well plates were gently washed once with ice-cold PBS, PBS was aspirated and ice-cold RIPA plus cocktail buffer (RIPA-200μL per well, with 200 mM Na_3_VO_4_, 500 mM NaF, 100 mM Na_4_P_2_O_4_) was added to scrape adherent cells off the plates or dishes using a cold plastic cell scraper, then the cell suspension was transferred into a pre-cooled microcentrifuge tube. Cell suspension was centrifuged in a microcentrifuge at 4°C for 10 mins at 4,000 g, supernatant was aspirated and placed in fresh ice-cooled tubes. BCA commercial kits was used to uniform the concentration of proteins by adding loading buffer and PBS. 10% separating gels and 5% stacking gels were prepared. Samples were loaded containing equal amounts of protein 50 μg/lane protein from cell lysate prepared in sample buffer into SDS-PAGE wells. Fill the electrophoresis apparatus with 1X running buffer with 100V for 30min and then 120V for 50mins. An equal amount of protein in each sample was subjected to sodium dodecyl sulphate‐polyacrylamide gel electrophoresis gels and then transferred to polyvinylidene fluoride (PVDF) membranes. After blocking with 5% BSA for 2 hours, membranes were incubated with the primary antibodies against ALDH1A3 (1:5000) overnight at 4°C and GAPDH (1:1000) was assessed as a loading control. After washing with TBST for three times, the membranes were incubated for 2 hours with the secondary antibodies. The protein bands were measured using Image-Pro plus 6.0 software (Media Cybernetics).

### 2.8 Glucose-stimulated insulin secretion

Before the test of GSIS, the MIN-6 cells were grown in 24-well plates and were treated with different concentrations of BBR () for 24 hours. Then the cells were rinsed twice and incubated with Krebs–Ringer bicarbonate (KRB) buffer containing 115 mM NaCl, 5 mM KCl, 24 mM NaHCO3, 2.5 mM CaCl2, 1 mM MgCl2, 10 mM HEPES, 2.8 mM glucose and 0.1% BSA for 60 min. After equilibration for 1 hour, cells were treated with 27 mM glucose in KRB buffer at 37°C for 30 mins, in the presence of various concentrations of BBR for another 30 mins. Supernatants were collected and insulin levels were detected by an in-house insulin ELISA kit. For the insulin detection, the insulin secretion was normalized by total insulin extracted from RIPA. 96 well plates were coated with 1/500 diluted anti-Insulin + proinsulin antibody-D6C4 (Abcam, ab8303) in PBS (50 μL/well) overnight at 4°C. Then the coated plates were blocked with blocking buffer (3% Bovine Serum Albumin (BSA) in PBS) (200 μL/well) for 1.5 hour at RT. Samples were diluted in blocking buffer for 10 times. The diluted samples (100 μL/well) were incubated with 96 well plates for 2 hours at RT. Detection antibody D3E7, (Abcam, ab8305) was diluted by 1/2000 with blocking buffer and incubated for 2 hours at RT (50 μL/well). 3,3′,5,5′-Tetramethylbenzidine (TMB) (100 μL/well) was incubated for 15 min at RT to visualize antibody reactivity and stopped by 2N H_2_SO_4_. Three-times of washing with PBS was required after each step of incubation. The absorbance was measured at 450nm (Tecan).

### 2.9 Statistical analysis

Data are presented as the mean ± SD. Between two groups, analyses were performed using t-test. Multiple group comparisons and analyses were performed based on a one-way ANOVA, followed by a *post hoc* Bonferroni test, as appropriate. A value of *p*< 0.05 was considered statistically significant and *p*< 0.01 was considered significantly different. Statistical analyses were carried out using GraphPad Prism 10 Software (USA).

## 3 Results

### 3.1 BBR exhibits apoptosis in MIN-6 cell apoptosis at high concentractions

**Figure 1:**
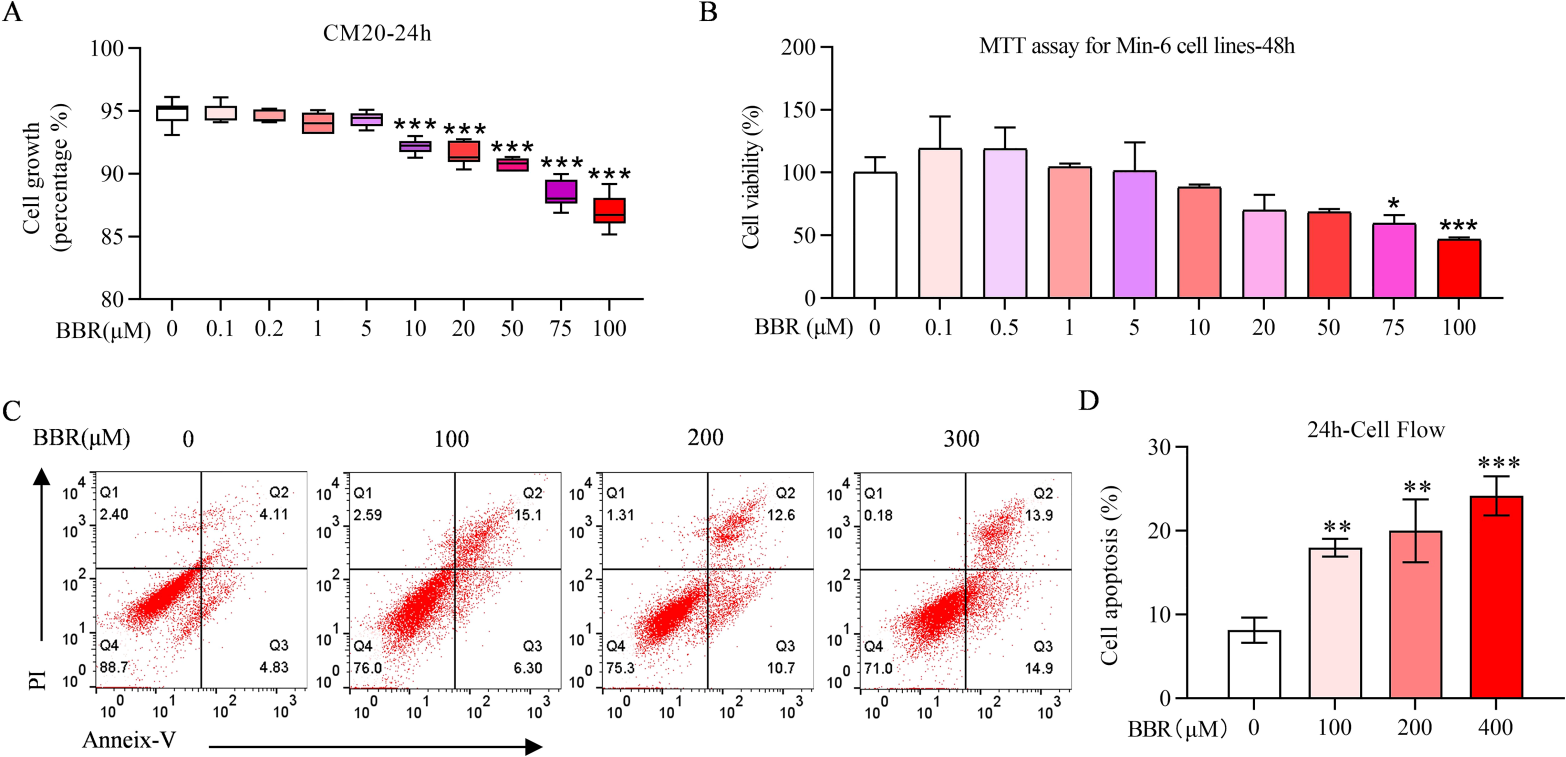
BBR exhibits apoptosis in MIN-6 cell apoptosis at high concentractions. Effect of BBR treatment on MIN-6 cells growth in various concentrations detected by CM20 with BBR on various concentrations for 24 hours **(A)** Cell viability detected by MTT experiments on MIN-6 cells under the pretreatment with BBR on various concentrations for 48 hours **(B)**. Cell apoptosis detected by cell flow cytometry **(C)**. Quantitative plot of cell flow cytometry **(D)**. All data were expressed as Mean ± S.D. Statistical difference was analysed by two-way ANOVA. *p < 0.05 or **p < 0.01 or ***p < 0.001 comparing with normal group.

BBR had a promoting effect on cell proliferation at low concentrations, but at high concentrations, the promoting effect on cell proliferation was diminished, and an inhibitory effect was observed using CM20 with 24-hour treatment of BBR up to 100 µM (**Figure 1A**). MTT results showed the same tendency (**Figure 1B**), which was verified by Flow cytometry (**Figures 1C, D**), suggesting high concentrations or long-term treatment of BBR potentially induced apoptosis or growth arrest.

### 3.2 BBR exhibits no significant effect on proliferation at low concentrations

**Figure 2:**
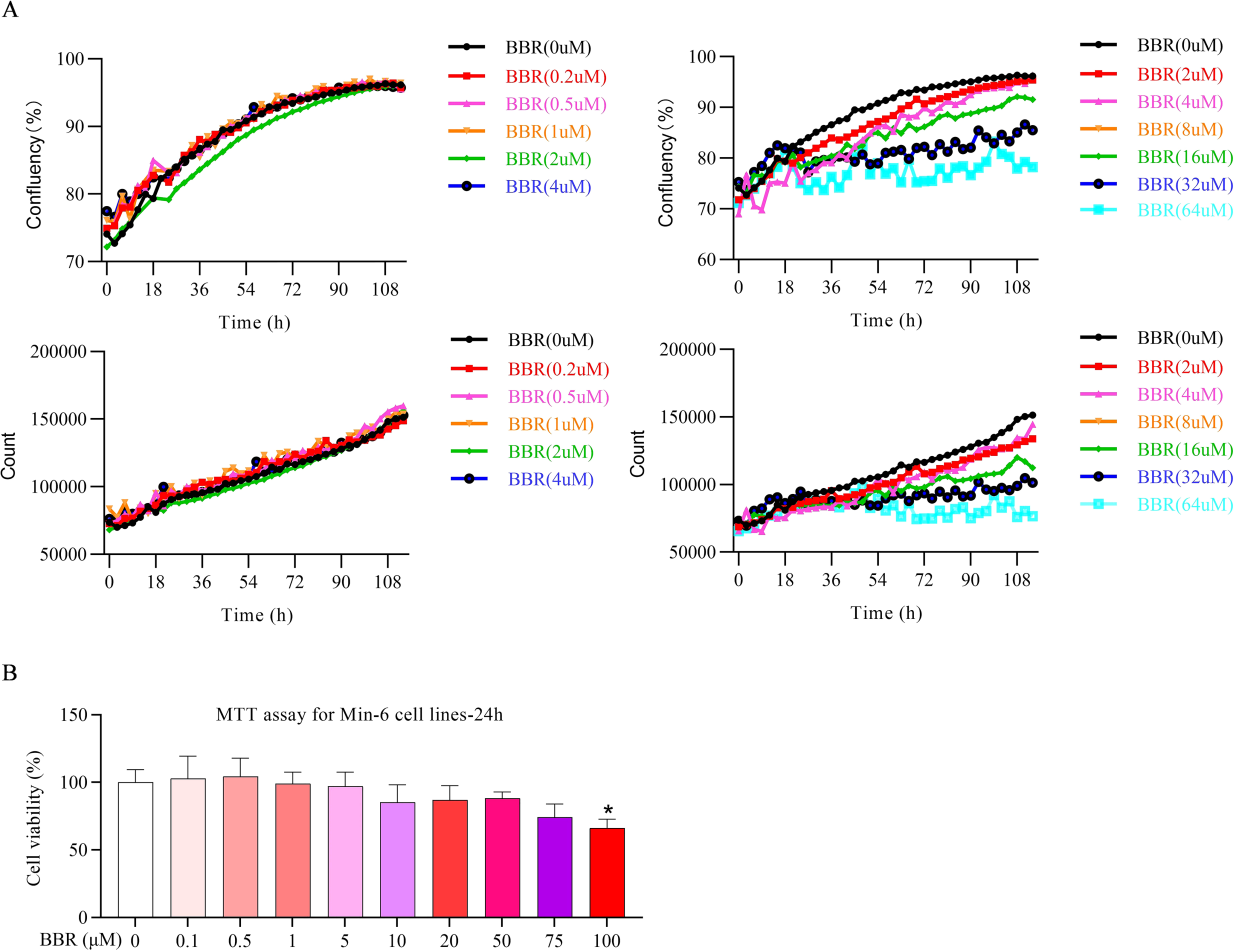
BBR exhibits no significant effect on proliferation at low concentrations. Effect of BBR treatment on MIN-6 cells growth at various concentration levels detected by CM20 with time durations **(A)**, Cell viability detected by MTT experiments on MIN-6 cells under the pretreatment with BBR on various concentrations **(B)**. All data were expressed as Mean ± S.D. Statistical difference was analysed by two-way ANOVA, *p < 0.05 comparing with normal group.

At lower concentration ranges, BBR exerted no obvious influences on cell proliferation in both short- and long-term treatment (**Figure 2A**), however, markedly reduced MIN-6 cells proliferation by long-term treatment (after 24-hour treatment) of BBR in a concentration dependent manner. MTT assay results indicated that BBR had a time-dependent effect on MIN-6 cell proliferation. After 24 hours of treatment across a wide concentration range, BBR had a relatively mild impact on cell growth. However, after 48 hours, the inhibitory effect on proliferation was more pronounced (**Figure 2B**). Therefore, in the following experiments, we chose 24h period and started lower concentrations of BBR to explore its efficacy on the cell dedifferentiation.

### 3.3 BBR reverses β-cell dedifferentiation induced by FoxO1 inhibitor

**Figure 3:**
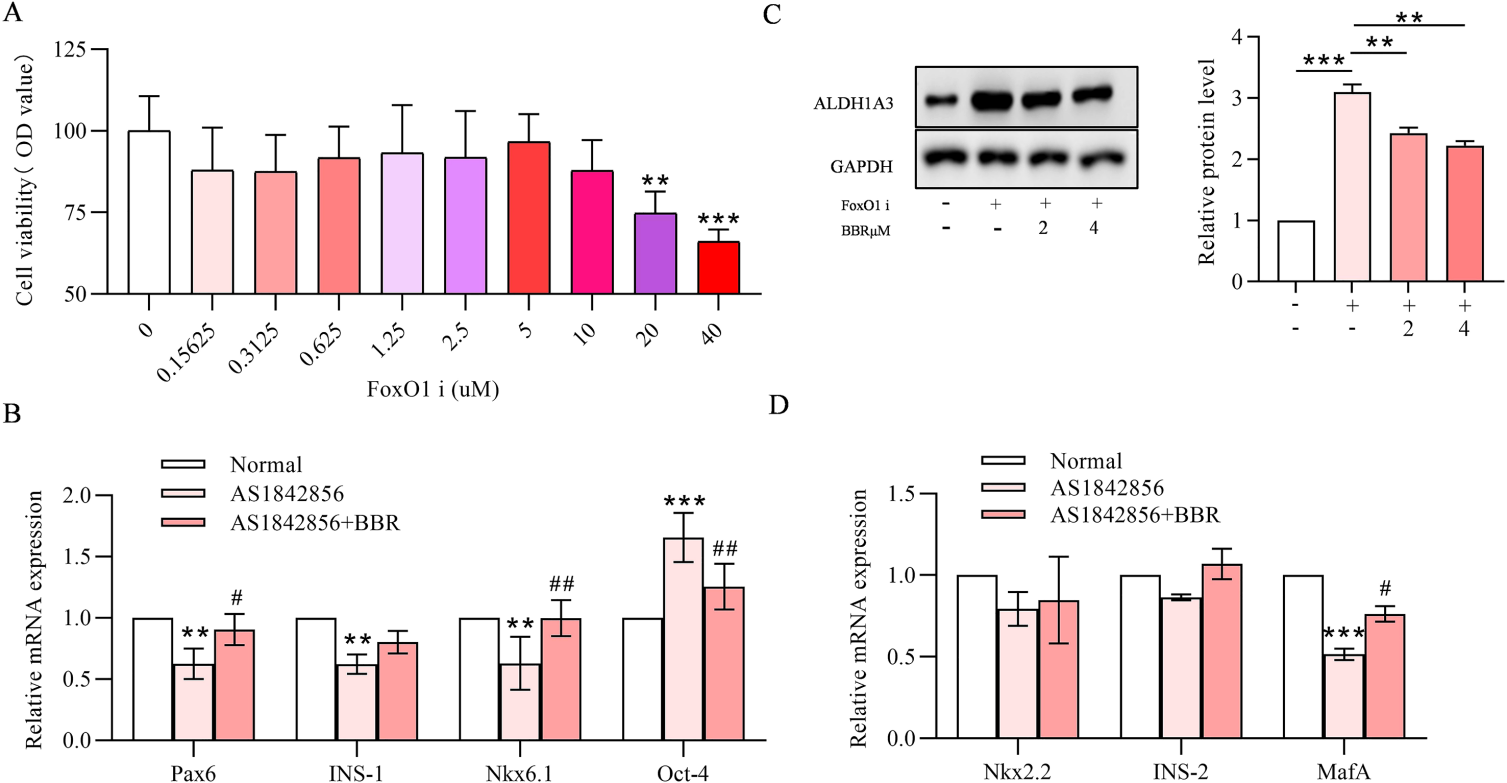
BBR reverses β-cell dedifferentiation induced by FoxO1 inhibitor. Cell viability was measured by MTT assay with different concentrations of FoxO-1 Inhibitor **(A)**. The relative RNA expression levels with or without the FoxO1 inhibitor **(B)**. ALDH1A3 protein expression detected by western blot and quantitative analysis with internal quantitative control of GAPDH **(C)**. ALDH1A3 protein expression level detected by immunofluorescence **(D)**. Scale bars data were expressed as mean ± S.D. Statistical difference was analysed by two-way ANOVA. **p < 0.01 or ***p < 0.001 comparing with normal group. ^#^*p* < 0.05 or ^##^*p* < 0.01 comparing with model group.

MIN-6 cells were treated with FoxO1 inhibitor (AS1842856) at various concentrations (0-40 µM) for 24 hours, and MTT assay was used to identify non-toxic concentrations on MIN-6 cells (20 and 40 µM) (**Figure 3A**). The AS1842856 (10 µM) was used to evaluate the efficacy of BBR on islet cell redifferentiation. The expression of β-cell differentiation-related genes, such as Pax6, INS-1, INS-2, MafA and Nkx6.1, were decreased under 24 h FoxO1 inhibitor treatment, comnfirming dedifferentiation induced by FoxO1 inhibition. Co-treatment with BBR restored the expression of these genes (**Figures 3B, D**). Additionally, immunofluorescence of ALDH1A3 was expressed in dedifferentiated pancreatic β cells. ALDH1A3 was also expressed in pancreatic β cells that have lost key functional features (21), and ablating ALDH1A3 in diabetic animals improves β-cell function and maturity, resulting in lower glycemia and higher insulin secretion (22). In **Figure 3C**, results showed that ALDH1A3 expression was increased by FoxO1 inhibitor, which was in line with previous data (23). The expression levels of ALDH1A3 were reversed with the co-treatment with BBR in a concentration dependent manner (**Figure 3C**).

### 3.4 BBR recoverses β-cell GSIS impaired by the FoxO1 inhibitor

**Figure 4:**
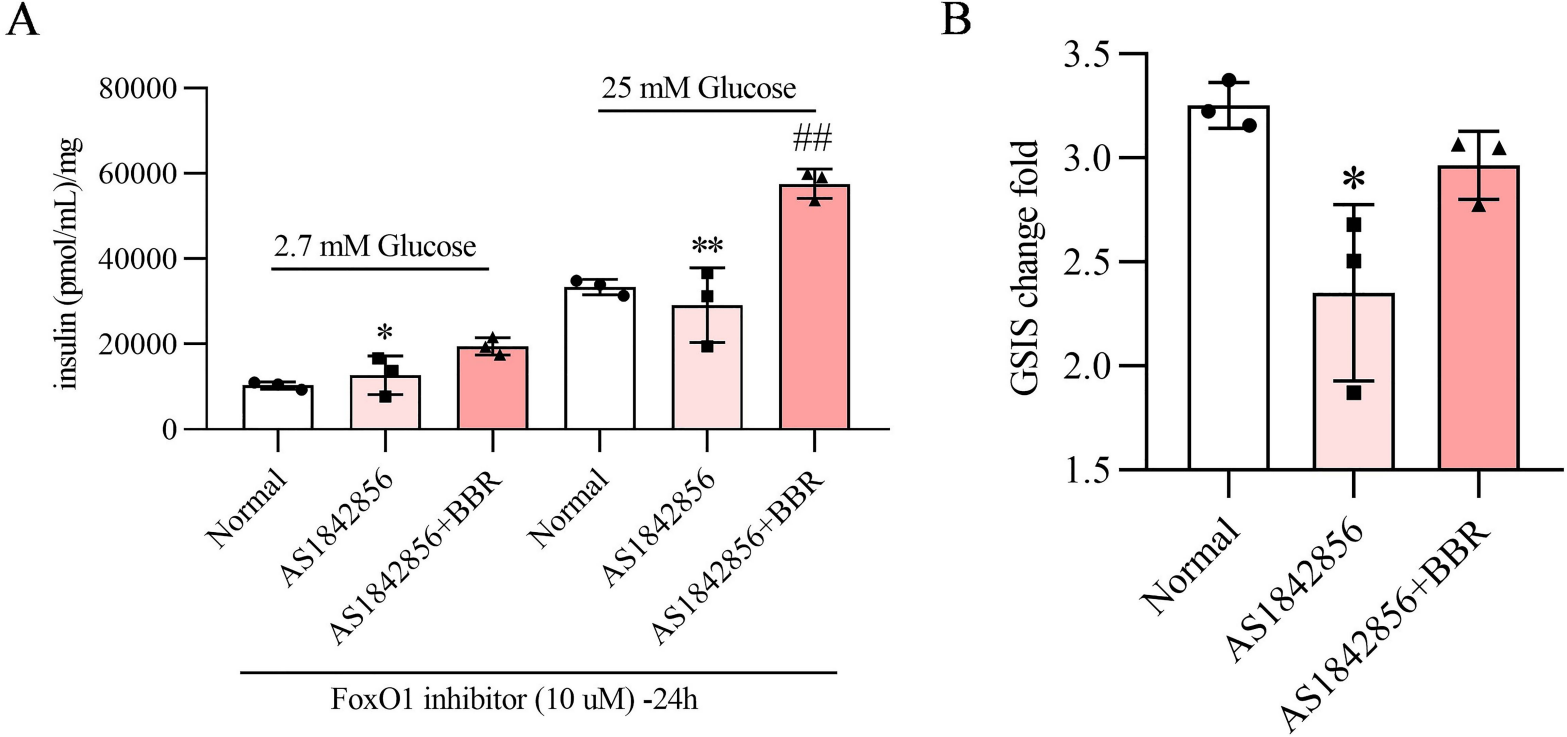
BBR recoverses β-cell GSIS impaired by the FoxO1 inhibitor.FoxO-1 inhibitor treatment effects on GSIS were reversed by co-treatment of BBR **(A)** GSIS change fold is increased by co-treatment of BBR **(B)**. Scale bars data were expressed as mean ± S.D. Statistical difference was analysed by two-way ANOVA. *p < 0.05 or **p < 0.01 comparing with normal group. ^#^*p* < 0.05 or ^##^*p* < 0.01 comparing with model group.

To investigate the effects of BBR on GSIS in β cells, particularly under conditions of stress induced by FoxO1 inhibitor, MIN-6 cells were treated by FoxO1 inhibitor with or without BBR. Three treatment groups were established: cells treated with vehicle; FoxO1 inhibitor group; FoxO1 inhibitor + BBR treatment group (4 µM) for 24 hours. FoxO1 inhibitor induced dedifferentiation and impaired GSIS caused by a low-glucose medium (2.7 mM glucose) for 1 hour then by a high-glucose medium (25 mM glucose) for another 1 hour. The impaired GSIS was reversed by the pretreatment with BBR (**Figure 4**).

### 3.5 MIN-6 dedifferentiation induced by H_2_O_2_ is reversed by BBR

**Figure 5:**
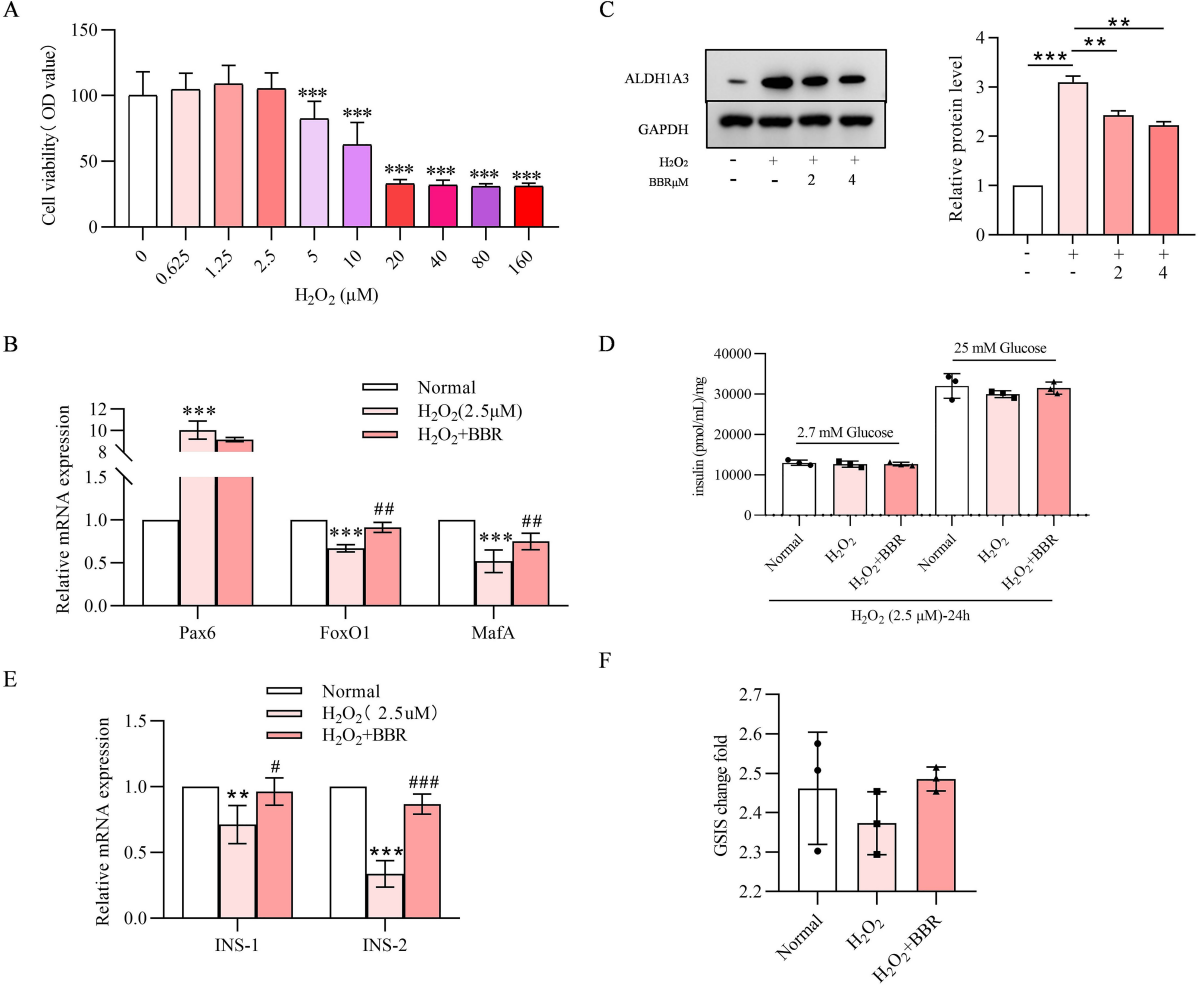
MIN-6 dedifferentiation induced by H_2_O_2_ was reversed by BBR. Cell viability detected by MTT experiments on MIN-6 cells under the treatment of H_2_O_2_ on various concentrations **(A)**.BBR effects on the relative RNA expression levels on the condition of H_2_O_2_ treatment **(B)**. ALDH1A3 protein expression level detected by western blot and quantitative analysis of ALDH1A3 **(C)**. H_2_O_2_ treatment effects on GSIS can be reversed by co-treatment of BBR **(D)**. BBR effects on the insulin secretion relative RNA expression levels on the condition of H_2_O_2_ treatment **(E)**. GSIS change fold is increased by co-treatment of BBR **(F)**. All data were expressed as Mean ± S.D. Statistical difference was analysed by two-way ANOVA. **p < 0.01 or ***p < 0.001 comparing with normal group. ^#^*p* < 0.05 or ^##^*p* < 0.01 or ^###^*p* < 0.001 comparing with model group.

A reduction in viability was observed at H_2_O_2_ concentrations >5 µM (**Figure 5A**). Gene expression level of Pax6 was increased under 2.5 µM H_2_O_2_ treatment, and FoxO1, MafA, INS-1 and INS-2 were decreased, suggesting dedifferentiation and impaired insulin secretion (**Figures 5B, E**). BBR (4 µM) co-treatment group for 24 hours reversed these expressions. Protein expression of ALDH1A3 was increased by H_2_O_2_ treatment, further illustrating the dedifferentiated β-cell state, and it was reduced by BBR in a concentration dependent manner (**Figure 5C**). Three treatment groups were established: cells treated with vehicle; H_2_O_2_ (2.5 µM) treatment group; H_2_O_2_ (2.5 µM) + BBR treatment group (4 µM) for 24 hours. H_2_O_2_ induced impaired GSIS, and the impaired GSIS was reversed by the pretreatment with BBR (**Figures 5D, F**).

### 3.6 FFAs induced dedifferentiation is reversed by BBR

**Figure 6:**
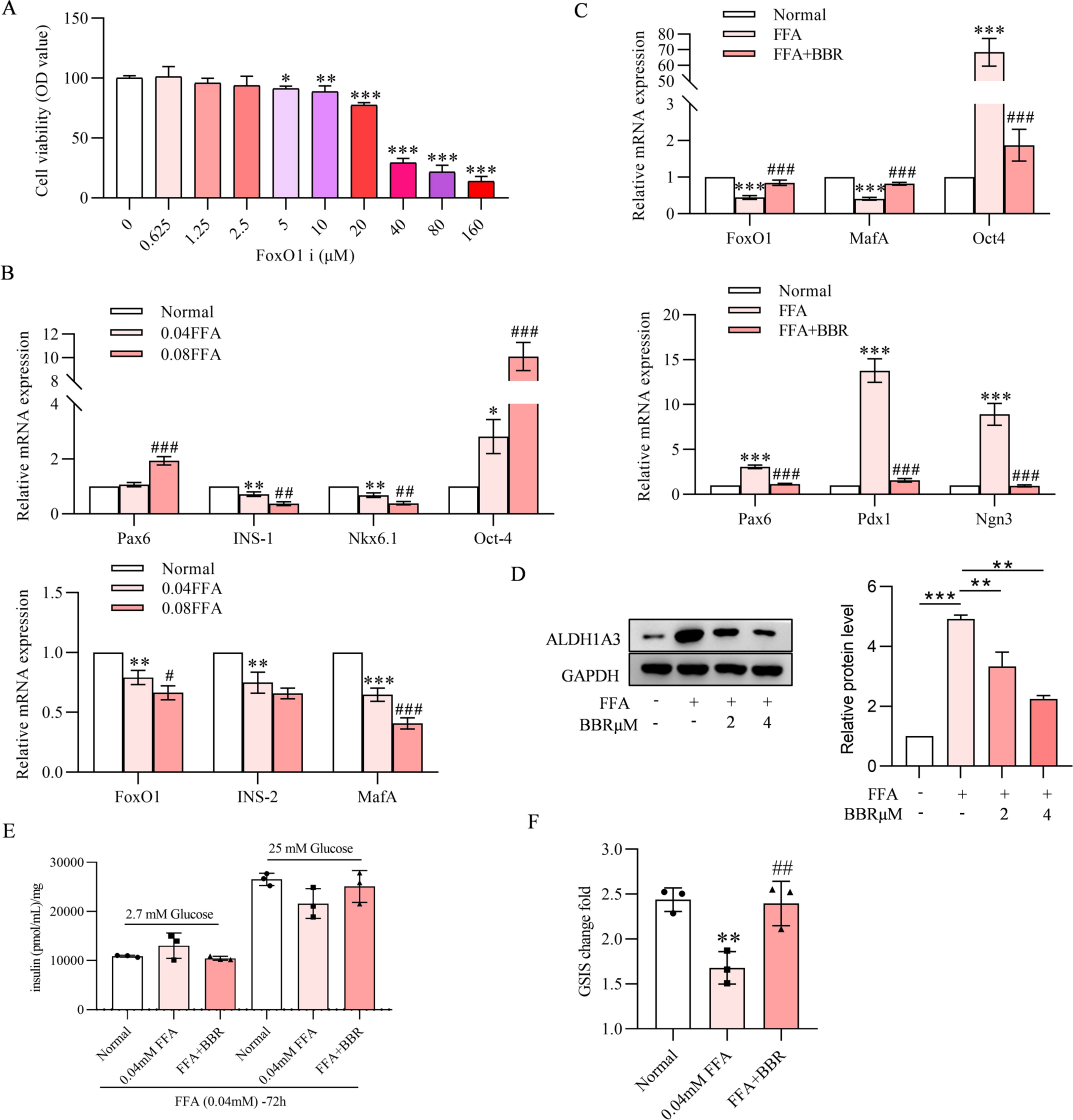
FFAs induced dedifferentiation is reversed by BBR. Cell viability detected by MTT experiments on Ins-1 cells under the treatment of FFAs on various concentrations **(A)**. BBR effects on the relative RNA expression levels on the condition of FFAs treatment **(B, C)**. ALDH1A3 protein expression level detected by western blot and quantitative analysis of ALDH1A3, the ALDN1A3 protein’s level was standardized to that of GAPDH **(D)**. FFAs treatment effects on GSIS can be reversed by co-treatment of BBR **(E)**. GSIS change fold is increased by co-treatment of BBR **(F)**. Scale bars data were expressed as mean ± S.D. Statistical difference were analysed by two-way ANOVA. *p < 0.05 or **p < 0.01 or ***p < 0.001 comparing with normal group. ^#^*p* < 0.05 or ^##^*p* < 0.01 or ^###^*p* < 0.001 comparing with model group.

Excessive exposure to FFAs may lead to mitochondrial dysfunction in pancreatic β cells, which contributes to the dedifferentiation of β cells involving in a loss of specialized features, functions, gene expression and cell identity. In this experiment, BBR effectively restored MIN-6 cells dedifferentiation exposed to FFAs and contribute to the reversal of dedifferentiation by preserving the cells’ metabolic and functional characteristics. FFAs for 24 hours decreased cell viability (**Figure 6A**). Gene expression level of Pax-6 and Oct-4 were increased by FFAs in a concentration dependent manner. Other insulin-related genes and cell identity relevant genes like INS-1, Nkx 6.1 FoxO1, INS-2 and MafA were decreased with FFAs concentration dependent manner, suggesting MIN-6 cells dedifferentiation was induced by 0.04 and 0.08 µM of FFAs treatment for 24 hours (**Figure 6B**). These genes changes induced by FFAs were reversed by pretreatment with BBR (**Figure 6C**). Increased ALDH1A3 protein expression induced by FFAs further confirmed the dedifferentiation state of MIN-6 cells, which was decreased by BBR in a concentration dependent manner (**Figure 6D**). Our data demonstrated that 0.04 µM FFAs treatment for 72h impaired the GSIS for MIN-6, and pretreatment with BBR for 24-hour showed detectable changes on MIN-6 cells GSIS (**Figures 6E, F**).

### 3.7 BBR reversed H_2_O_2_, FFAs and FoxO1-induced β-cell dedifferentiation through NF-κB signaling pathway

**Figure 7:**
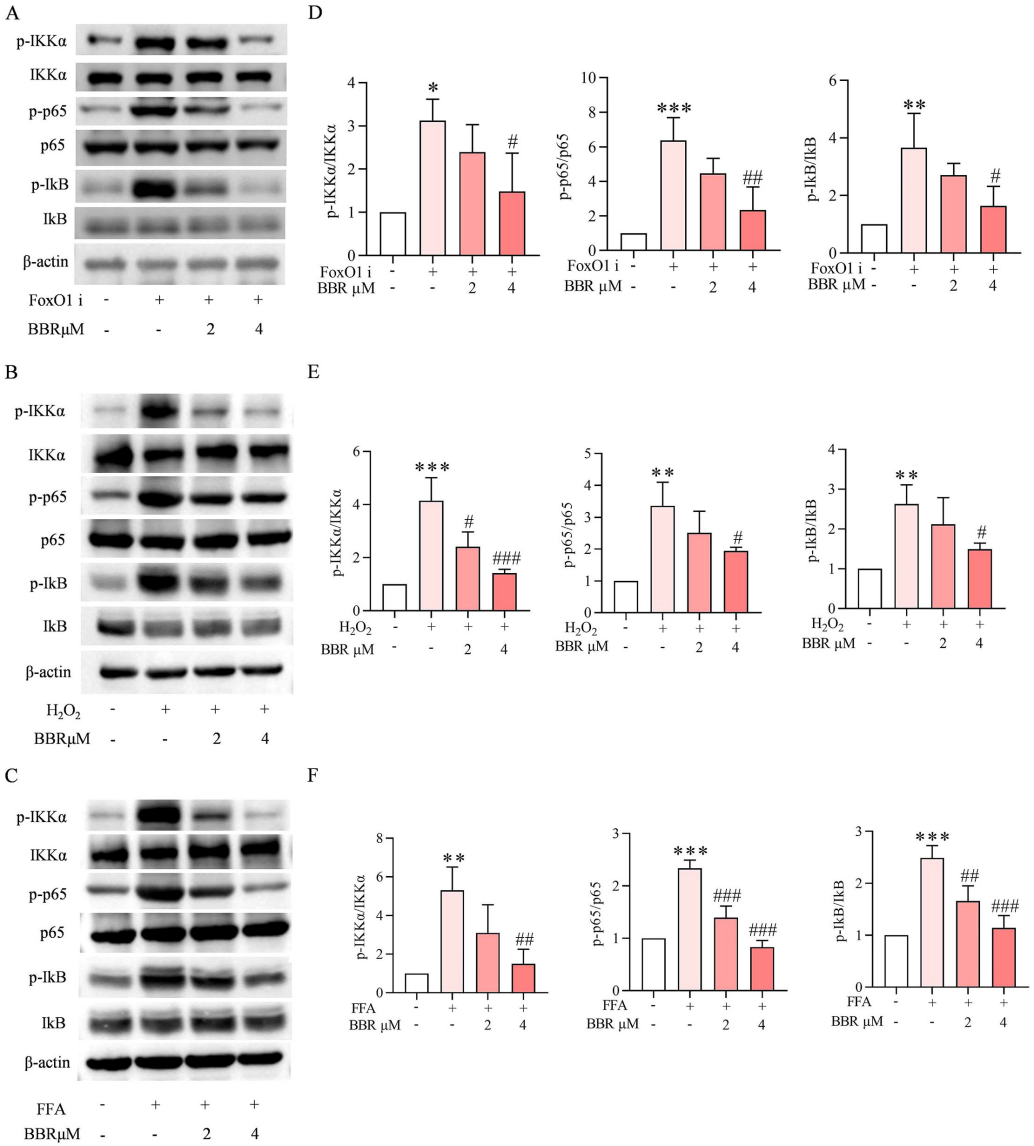
BBR reduced β-cell dedifferentiation through NF-κB signaling pathway. Molecular docking NF-κB with BBR. The western blot of p-IKKα; IKKα; p-p65; p65; p-IkB; IkB and β-actin expression levels in the presence of FoxO1 inhibitor, H_2_O_2_ and FFAs **(A–C)**. Quantitative analysis of p-IKKα/IKKα, p-p65/p65, p-IkB/IkB in the presence of FoxO1 inhibitor **(D)**. Quantitative analysis of p-IKKα/IKKα, p-p65/p65 and p-IkB/IkB in the presence of H_2_O_2_**(E)**. Quantitative analysis of p-IKKα/IKKα, p-p65/p65, p-IkB/IkB in the presence of FFAs **(F)**. All proteins were standardized to that of β-actin. Scale bars data were expressed as mean ± S.D. Statistical difference was analysed by two-way ANOVA. *p < 0.05 or **p < 0.01 or ***p < 0.001 comparing with normal group. ^#^*p* < 0.05 or ^##^*p* < 0.01 or ^###^*p* < 0.001 comparing with model group.

**Figure 8:**
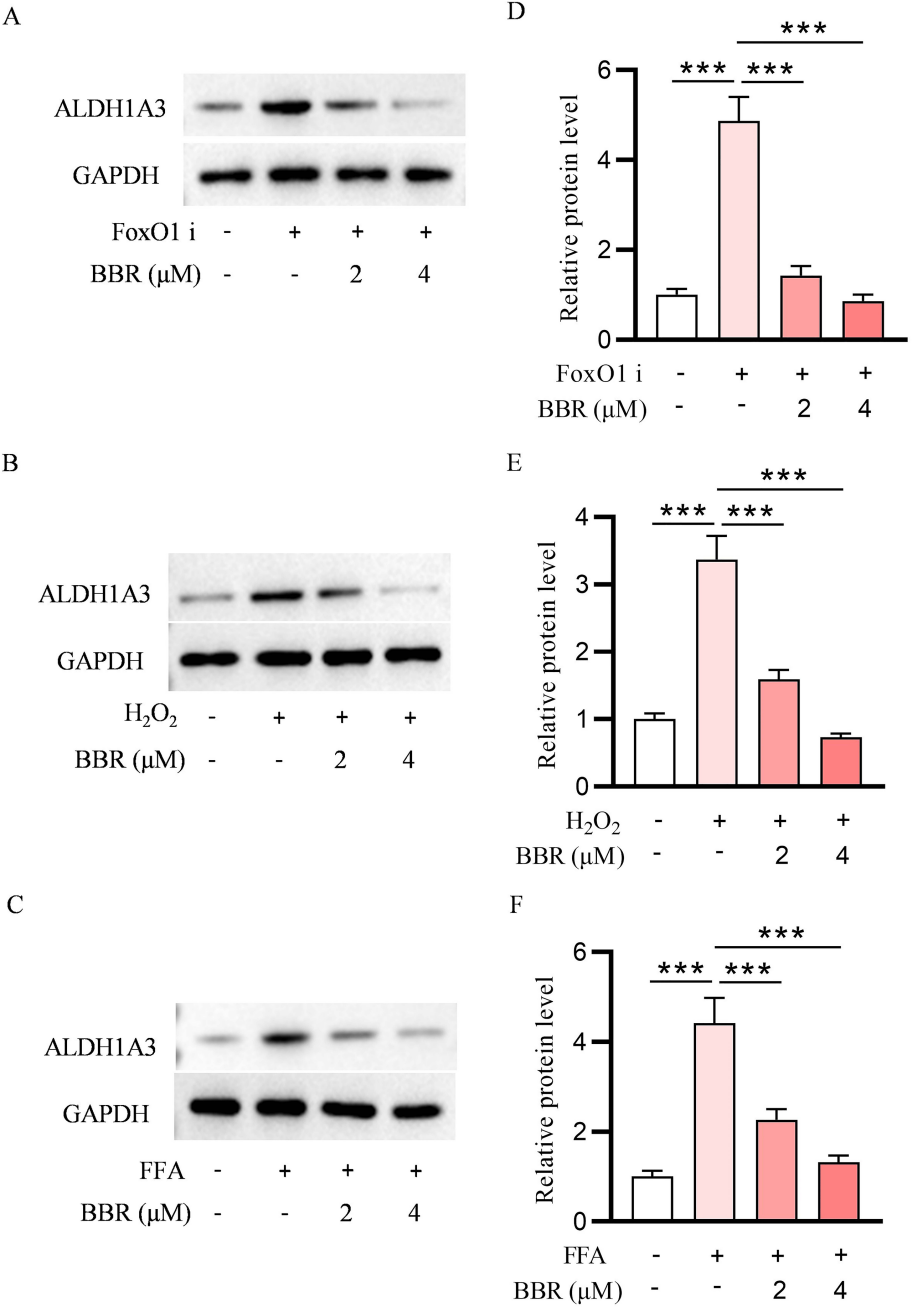
INS-1 dedifferentiation induced by FoxO1 inhibitor, FFA and H_2_O_2_ was reversed by BBR. ALDH1A3 protein expression level detected by immunofluorescence under the treatment of FoxO1 inhibitor with or without BBR **(A)**, ALDH1A3 protein expression level detected by immunofluorescence under the treatment of H_2_O_2_ with or without BBR **(B)**, ALDH1A3 protein expression level detected by immunofluorescence under the treatment of FFA with or without BBR **(C)**. Quantitative analysis of ALDH1A3 in the presence of FoxO1 inhibitor **(D)**, Quantitative analysis of ALDH1A3 in the presence of H_2_O_2_**(E)**, Quantitative analysis of ALDH1A3 in the presence of FFA **(F)**. Scale bars data were expressed as mean ± S.D. Scale bars data were expressed as mean ± S.D. Statistical difference was analysed by two-way ANOVA. ***p < 0.001 comparing with normal group.

Our western blot data showed that free fatty acids, H_2_O_2_, FFAs and FoxO1 inhibitor treatment induced the over-stimulated NF-κB signaling pathway, the expression levels of p-IKKα, p-p65, p-IkB were increased in the presence of FoxO1 inhibitor, while the increased levels of NF-κBs were reversed by the pre-treatment of BBR in a concentration dependent manner (**Figures 7A–C**). To investigate the role of the NF-κB signaling pathway in oxidative stress– and lipotoxicity-induced β-cell dedifferentiation, INS-1 cells were treated with H_2_O_2_, FFAs, or a FoxO1 inhibitor in the presence or absence of BBR. Western blot analysis revealed that all three treatments markedly increased the phosphorylation of IκBα and p65, indicating activation of the NF-κB pathway (**Figures 8A–C**). This activation was accompanied by a significant reduction in key β-cell identity markers including Pdx1, MafA, and Insulin, and a concomitant upregulation of dedifferentiation markers such as Ngn3 and ALDH1A3. Co-treatment with BBR effectively attenuated NF-κB activation, as evidenced by reduced phosphorylation of IκBα and p65. Importantly, BBR treatment restored the expression of β-cell identity markers and suppressed dedifferentiation markers across all three models. These effects were comparable to those observed with the NF-κB inhibitor BAY 11-7082, further supporting the involvement of NF-κB signaling in this process. Collectively, these findings indicate that BBR reverses β-cell dedifferentiation induced by oxidative stress, lipotoxicity, and FoxO1 inhibition, at least in part through suppression of NF-κB signaling.

### 3.8 BBR reverses ALDH1A3 upregulation in dedifferentiated INS-1 cells

To assess the effect of BBR on INS-1 cells dedifferentiation, we evaluated the expression of ALDH1A3, in INS-1 cells under different stress conditions using immunofluorescence staining. Treatment with the FoxO1 inhibitor led to a marked increase in ALDH1A3 expression compared to the normal group, indicating the induction of a dedifferentiated phenotype (**Figure 8A**). Notably, co-treatment with BBR significantly reduced ALDH1A3 levels, suggesting a reversal of dedifferentiation. Similarly, ALDH1A3 expression was elevated in cells exposed to H_2_O_2_ (**Figure 8B**) or FFAs (**Figure 8C**), and BBR treatment effectively attenuated these increases.

Quantitative analysis confirmed these findings: ALDH1A3 levels were significantly upregulated by FoxO1 inhibitor, H_2_O_2_, and FFA treatment (**Figures 8D–F**), while BBR significantly suppressed this upregulation. These data collectively indicate that BBR mitigates β-cell dedifferentiation induced by oxidative stress, lipotoxicity, or transcriptional deregulation.

### 3.9 BBR attenuates NF-κB pathway activation to prevent INS-1 cell dedifferentiation

**Figure 9:**
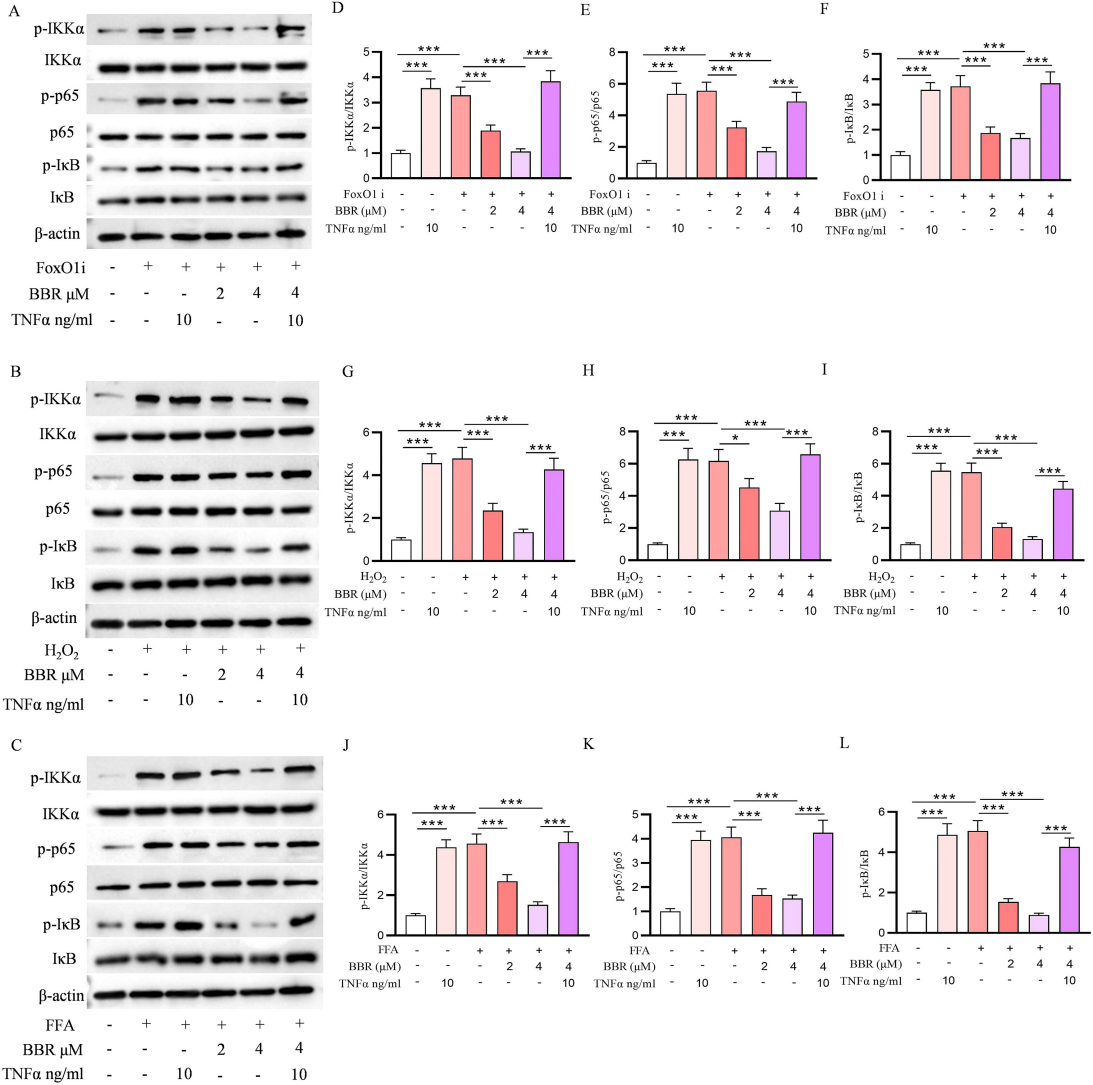
NF-κB antagonist confirms BBR acts through NF-κB signaling to prevent INS-1 dedifferentiation. Molecular docking NF-κB with BBR. The western blot of p-IKKα; IKKα; p-p65; p65; p-IkB; IkB and β-actin expression levels in the presence of FoxO1 inhibitor H_2_O_2_ and FFAs with or without NF-κB antagonist **(A–C)**. Quantitative analysis of p-IKKα/IKKα, p-p65/p65, p-IkB/IkB in the presence of FoxO1 inhibitor with or without NF-κB activator **(D–F)**. Quantitative analysis of p-IKKα/IKKα, p-p65/p65 and p-IkB/IkB in the presence of H_2_O_2_ with or without NF-κB antagonist **(G–I)**. Quantitative analysis of p-IKKα/IKKα, p-p65/p65, p-IkB/IkB in the presence of FFAs **(J–L)**. All proteins were standardized to that of β-actin. Scale bars data were expressed as mean ± S.D. Statistical difference was analysed by two-way ANOVA. *p < 0.05 or ***p < 0.001 comparing with normal group.

To investigate whether BBR prevents β-cell dedifferentiation through inhibition of NF-κB signaling, molecular docking analysis was first performed and revealed a potential binding interaction between BBR and NF-κB, suggesting a direct regulatory effect. We next examined the protein levels of key NF-κB pathway components by Western blot in INS-1 cells treated with FoxO1 inhibitor, H_2_O_2_, or FFAs, with or without an NF-κB agonist. As shown in **Figures 9A–C**, the phosphorylated forms of IKKα, p65, and IκB (p-IKKα, p-p65, and p-IκB) were significantly upregulated following treatment with FoxO1 inhibitor, H_2_O_2_, or FFAs, indicating pathway activation. These changes were effectively suppressed by co-treatment with an NF-κB antagonist, supporting the involvement of this signaling cascade in stress-induced dedifferentiation. Quantitative analyses confirmed that the ratios of p-IKKα/IKKα, p-p65/p65, and p-IκB/IκB were significantly increased in response to each stressor, and significantly decreased upon co-treatment with the NF-κB antagonist (**Figure 9**). These results demonstrate that inhibition of the NF-κB pathway can reverse the stress-induced activation, consistent with the protective effects observed with BBR.

### 3.10 NF-κB activator confirms BBR acts through NF-κB signaling to prevent β-cell dedifferentiation

To further validate that BBR preserves β-cell identity by inhibiting the NF-κB signaling pathway, we employed the NF-κB activator TNFα. INS-1 cells were treated with H_2_O_2_, FFAs, or a FoxO1 inhibitor, either alone or in combination with BBR, the NF-κB activator TNFα. Western blot analysis showed that treatment with H_2_O_2_, FFAs, or FoxO1 inhibitor markedly activated NF-κB signaling, as evidenced by increased phosphorylation of IκBα and p65. This activation was accompanied by downregulation of β-cell identity markers (Pdx1, MafA, Insulin) and upregulation of dedifferentiation markers (Ngn3, ALDH1A3), consistent with β-cell dedifferentiation. Treatment with TNFα alone further enhanced NF-κB activation and reversed the protective effects of BBR, reinstating the dedifferentiated phenotype. Interestingly, co-treatment with both BBR and TNFα did not yield additional effects beyond those of TNFα alone, suggesting that H_2_O_2_, FFAs, or FoxO1 inhibitor and TNFα modulate β-cell dedifferentiation via a shared mechanism centered on the NF-κB signaling pathway, and BBR reversed the overproduction of NF-κB signaling (**Figure 9**). Collectively, these results provide strong evidence that the protective effects of BBR against β-cell dedifferentiation—triggered by oxidative stress, lipotoxicity, and FoxO1 inhibition—are mediated, at least in part, through suppression of NF-κB signaling.

## 4 Discussion

β-cell death, dysfunction, and dedifferentiation are well-established contributors to the pathophysiology of T2DM (24). These processes lead to progressive β-cell failure and impaired insulin secretion, particularly in the context of obesity or prolonged hyperglycemia. Strategies aimed at preventing β-cell dedifferentiation or restoring β-cell identity could therefore offer significant therapeutic potential for delaying or reversing the onset of T2DM.

Both animal (8, 25–27) and human (28, 29) studies have demonstrated that β-cells revert to a dedifferentiated stage during diabetes progression, losing their specialized functions and adopting progenitor-like or alternate endocrine phenotypes, including α-like glucagon-producing cells. This transition reflects a loss of terminal β-cell identity and function, often referred to as β-cell dedifferentiation.

FoxO1, a forkhead box transcription factor, has emerged as a critical regulator of β-cell identity (30). It governs a complex transcriptional network essential for β-cell development and functional maintenance (31). In response to metabolic cues such as hyperglycemia, lipid accumulation, or inflammation, FoxO1 translocates from the cytoplasm to the nucleus, triggering downstream gene expression changes that compromise β-cell function (32). Specifically, FoxO1 regulates core β-cell identity markers such as Pdx1, MafA, Pax6, INS1, and INS2. A decrease in FoxO1 activity has been associated with the loss of β-cell identity and the acquisition of progenitor-like traits (33).

Talchai et al. (8) first described the phenomenon of β-cell dedifferentiation, later elaborated by Domenico et al. who defined dedifferentiated β-cells as those lacking insulin content but retaining endocrine features and progenitor markers (28). According to these criteria, approximately 40% of β-cells in diabetic models display a dedifferentiated phenotype, characterized by downregulation of FoxO1, Nkx6.1, and MafA, while a subset (~4%) becomes degranulated and metabolically inert (8). Experimental inactivation of key transcription factors in mice, such as MafA, leads to impaired GSIS despite preserved β-cell mass (29. 27, 34). In both db/db mice and early-stage human T2DM, loss of nuclear MafA represents one of the earliest molecular signatures of dysfunction (8, 35). Furthermore, FoxO1 ablation in adult mouse β-cells has been shown to induce expression of progenitor markers like Ngn3, Oct4, and Nanog, further supporting its role in maintaining β-cell maturity (19, 29, 36–38).

However, *in vitro* models that hindered progress into the identification of key downstream targets and potential inhibitors were not tested yet. In this report, *in vitro* short-term and long-term cell dedifferentiation models were established to investigate the mechanism of BBR action.

Oxidative stress and lipotoxicity, commonly induced by hyperglycemia and elevated FFAs, have been shown to impair β-cell function in part through the generation of ROS (39). ROS can suppress transcription factors like MafA, further impairing insulin secretion (16). We observed that treatment with H_2_O_2_ and FFAs significantly impaired GSIS and reduced expression of INS-1 and INS-2, while promoting dedifferentiation marker expression. This supports the notion that oxidative stress and lipid overload play central roles in β-cell failure during diabetes progression.

BBR, a natural isoquinoline alkaloid with established anti-diabetic and anti-inflammatory properties, demonstrated a robust ability to reverse β-cell dedifferentiation across all models tested. BBR treatment restored β-cell identity gene expression, reduced progenitor markers, and improved GSIS in MIN-6 cells subjected to H_2_O_2_, FFAs, or FoxO1 inhibition. Notably, BBR attenuated the upregulation of ALDH1A3, a marker previously associated with immature or dysfunctional β-cells (19, 20).

A key mechanistic insight from our study involves the NF-κB signaling pathway, a central mediator of cellular stress and inflammation. Chronic β-cell stress activates IKKα/β, leading to phosphorylation and degradation of IκBα, and subsequent nuclear translocation of p65, promoting transcription of proinflammatory and stress response genes. Our results revealed that H_2_O_2_, FFAs, and FoxO1 inhibitor all activated NF-κB signaling, as evidenced by increased phosphorylation of IKKα, IκBα, and p65. This was accompanied by β-cell dedifferentiation and GSIS impairment.

Importantly, BBR treatment effectively suppressed NF-κB activation in a concentration-dependent manner. Western blot showed decreased phosphorylation of IκBα and p65 following BBR co-treatment, coinciding with restored β-cell identity and reduced dedifferentiation markers. These effects were comparable to those induced by BAY 11-7082, a selective NF-κB inhibitor, indicating that BBR may exert its protective effects at least partly through NF-κB suppression.

While NF-κB has been implicated in promoting inflammation and insulin resistance, its role in β-cell dedifferentiation remains underexplored. Our findings highlight NF-κB as a potential therapeutic target in preserving β-cell identity and function, particularly under conditions of metabolic stress. Modulating this pathway may represent a novel avenue to prevent or reverse β-cell dedifferentiation and dysfunction in diabetes.

In the present study, we established both short-term and long-term *in vitro* models of β-cell dedifferentiation using H_2_O_2_, FFAs, and a FoxO1 inhibitor. Our data demonstrated that these stressors reduced β-cell viability, impaired GSIS, and decreased expression of key β-cell identity genes, while increasing progenitor-associated markers such as ALDH1A3 and Oct4—consistent with a dedifferentiated β-cell phenotype, implicated in β-cell dysfunction in diabetes. Our findings support previous reports suggesting that sustained NF-κB activation contributes to β-cell dedifferentiation and loss of insulin secretory function under metabolic stress. By attenuating NF-κB signaling, BBR appears to preserve β-cell identity and prevent dedifferentiation *in vitro*, even under oxidative and lipotoxic conditions. These results highlight a novel mechanism by which BBR may exert its β-cell protective effects—namely, through inhibition of the NF-κB pathway. Given the pivotal role of NF-κB in various diabetic pathologies, targeting this pathway may offer a promising strategy for the preservation or restoration of β-cell function. Further investigation *in vivo* will be necessary to confirm these protective effects of BBR and to determine its therapeutic potential for the treatment of T2DM.

The mechanism underlying BBR’s protective effect likely extends beyond NF-κB inhibition. BBR may also modulate upstream regulators of metabolism, redox homeostasis, and mitochondrial function, all of which warrant further exploration. Nevertheless, current data underscore the ability of BBR to modulate transcriptional programs involved in β-cell plasticity and identity maintenance. While this study demonstrates that BBR suppresses NF-κB activation to mitigate β-cell dedifferentiation, the effectors of NF-κB pathway like IL-6, TNF-α, or COX-2 have not been thoroughly analyzed. The other potencial mechanism is that BBR may also influence AMPK activation, ER stress responses, and mitochondrial function, all of which are known to contribute to β-cell health. These pathways have not been fully explored in the present study.

**Figure 10:**
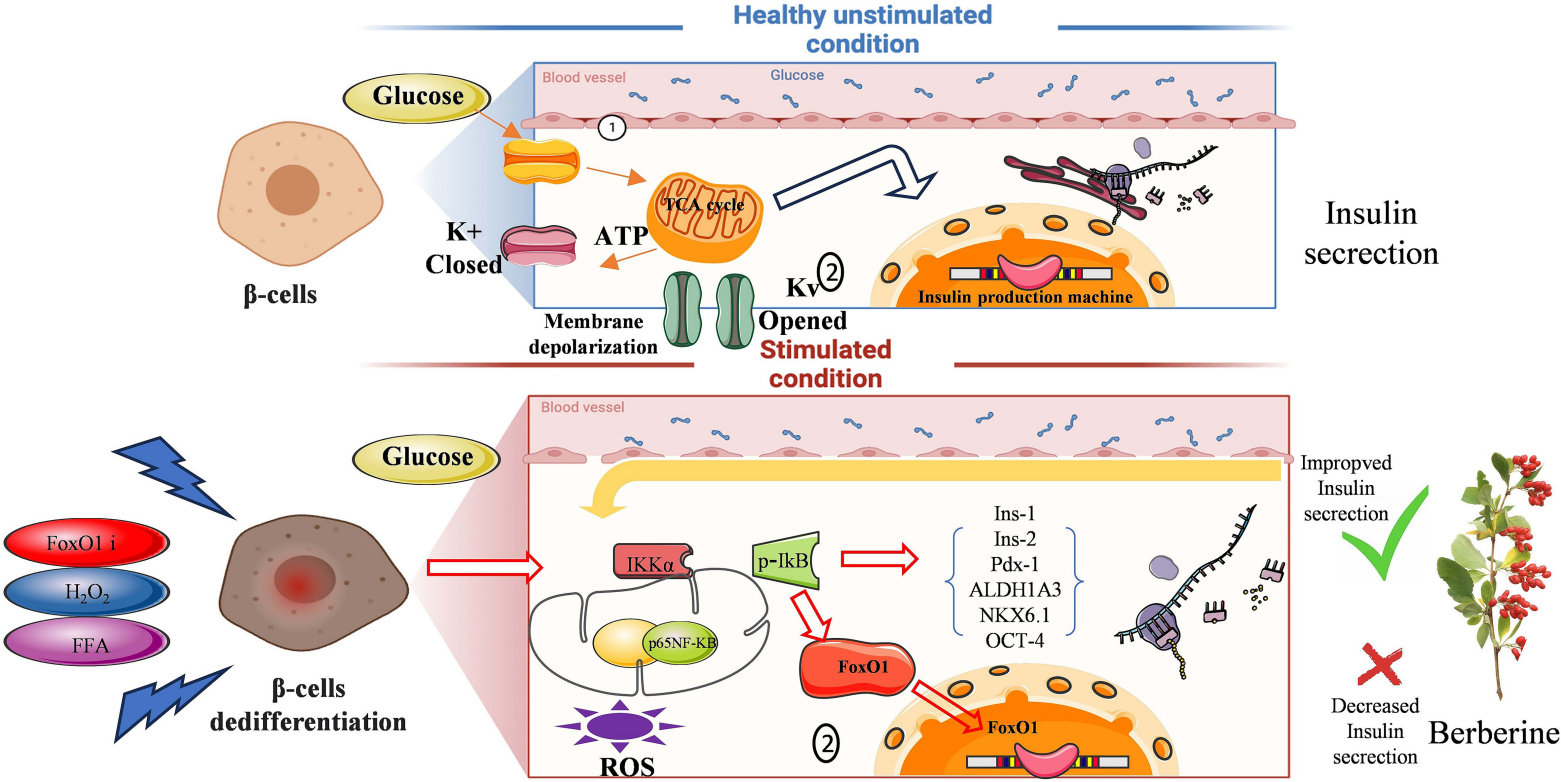
Mechanisms of BBR reversing cell dedifferentiation. This schematic illustrates the molecular mechanisms by which BBR preserves β-cell function and reverses dedifferentiation. Under pathological conditions, including FoxO1 inhibition, excessive oxidative stress (H_2_O_2_), and elevated free fatty acids (FFAs), β cells undergo dedifferentiation, leading to impaired glucose-stimulated insulin secretion (GSIS). These stressors activate the NF-κB signaling pathway, exacerbating β-cell dysfunction by decreasing insulin-1 and insulin-2 genes expressions and increasing ALDH1A3, OCT-4 expressions. BBR treatment counteracts these effects by inhibiting NF-κB overactivation, restoring FoxO1 activity, and mitigating oxidative stress and lipid toxicity. As a result, BBR prevents β-cell dedifferentiation, restores insulin secretion, and enhances β-cell responsiveness to glucose.

In conclusion, this study provides compelling evidence that oxidative stress, FoxO1 inhibition, and lipotoxicity promote β-cell dedifferentiation via activation of the NF-κB signaling pathway. BBR effectively reversed these changes, restoring β-cell identity and improving insulin secretion. These findings reveal a promising role for BBR in combating β-cell failure in T2DM and support its further development as a potential therapeutic agent. Future *in vivo* studies are necessary to validate these mechanisms and evaluate the long-term benefits of BBR in maintaining β-cell function and glycemic control (**Figure 10**).

## Data Availability

The datasets presented in this study can be found in online repositories. The names of the repository/repositories and accession number(s) can be found below: We have uploaded all the Raw data of Western blot.
